# Machine Learning-Driven Design and Optimization of Multi-Metal Nitride Hard Coatings via Multi-Arc Ion Plating Using Genetic Algorithm and Support Vector Regression

**DOI:** 10.3390/ma18153478

**Published:** 2025-07-24

**Authors:** Yu Gu, Jiayue Wang, Jun Zhang, Yu Zhang, Bushi Dai, Yu Li, Guangchao Liu, Li Bao, Rihuan Lu

**Affiliations:** 1 College of Mechanical Engineering, Shenyang University, Shenyang 110044, China; tongxun_e@126.com (J.Z.); 670003099@163.com (Y.Z.); bushi0509@126.com (B.D.); liugclxy@126.com (G.L.); 2 Laboratory of Research and Application of Multiple Hard Films, Shenyang University, Shenyang 110044, China; 3 Informatization Office, Shenyang University, Shenyang 110044, China; jiayue.wang@syu.edu.cn; 4 College of Intelligence and Informatics, Shenyang University, Shenyang 110044, China; w179yxl@syu.edu.cn; 5 School of Mechanical and Electrical Engineering, Qiqihar University, Qiqihar 161000, China; lwjxs2019@163.com; 6 National Engineering Research Center for Equipment and Technology of Cold Rolled Strip, Yanshan University, Qinhuangdao 066004, China; lrh@ysu.edu.cn

**Keywords:** machine learning, small sample problem, multi-arc ion plating, XN coating, hardness

## Abstract

The goal of this study is to develop an efficient machine learning framework for designing high-hardness multi-metal nitride coatings, overcoming the limitations of traditional trial-and-error methods. The development of multicomponent metal nitride hard coatings via multi-arc ion plating remains a significant challenge due to the vast compositional search space. Although theoretical studies in macroscopic, mesoscopic, and microscopic domains exist, these often focus on idealized models and lack effective coupling across scales, leading to time-consuming and labor-intensive traditional methods. With advancements in materials genomics and data mining, machine learning has become a powerful tool in material discovery. In this work, we construct a compositional search space for multicomponent nitrides based on electronic configuration, valence electron count, electronegativity, and oxidation states of metal elements in unary nitrides. The search space is further constrained by FCC crystal structure and hardness theory. By incorporating a feature library with micro-, meso-, and macro-structural characteristics and using clustering analysis with theoretical intermediate variables, the model enriches dataset information and enhances predictive accuracy by reducing experimental errors. This model is successfully applied to design multicomponent metal nitride coatings using a literature-derived database of 233 entries. Experimental validation confirms the model’s predictions, and clustering is used to minimize experimental and data errors, yielding a strong agreement between predicted optimal molar ratios of metal elements and nitrogen and measured hardness performance. Of the 100 Vickers hardness (HV) predictions made by the model using input features like molar ratios of metal elements (e.g., Ti, Al, Cr, Zr) and atomic size mismatch, 82 exceeded the dataset’s maximum hardness, with the best sample achieving a prediction accuracy of 91.6% validated against experimental measurements. This approach offers a robust strategy for designing high-performance coatings with optimized hardness.

## 1. Introduction

Multi-metal nitride hard coatings are widely used in industrial applications like cutting tools, molds, and high-performance surface engineering due to their excellent wear, corrosion resistance, and high-temperature stability [1,2,3,4,5,6,7]. They can withstand extreme conditions, being crucial in environments with mechanical stress, high temperatures, and corrosive elements. However, designing these coatings for optimal performance is challenging. This is mainly because of the complexity of their composition systems and intricate microstructures [8,9,10].

Traditional material development mainly relies on trial-and-error experiments, which are time- and resource-consuming, involving repeated synthesis and testing. Guided by macro-, meso-, and micro-level theories, these approaches lack integration among theoretical frameworks [11,12]. The gap between theoretical predictions and experimental validation causes inefficiencies in development, slowing down new material discovery and optimization. In multi-arc ion plating for multi-metal nitride coatings, the challenge is greater due to the vast compositional search space. Considering multiple metallic elements and process parameters like temperature, pressure, and deposition time, the system complexity grows exponentially, making it hard to explore all potential configurations [13,14].

To tackle these challenges, researchers are exploring computational approaches to complement traditional experiments. With the rising significance of computational materials science, especially materials informatics and data mining, material design has entered a new era. These techniques allow for more efficient exploration of material composition and process spaces, reducing the dependence on pure experimentation [15,16,17,18]. Recent computational method advances have transformed material discovery and optimization. A key development area is applying machine learning (ML) and algorithms like genetic algorithms (GAs) to materials science. ML models using large datasets can predict material properties from input parameters such as composition and processing conditions [19,20,21,22,23,24,25,26]. Using these models, researchers can predict new material behavior before synthesis, saving time and resources.

For example, genetic algorithms (GAs) are widely used in material optimization, especially for finding the best combination of compositional and process variables. GAs mimic natural selection, using crossover, mutation, and selection to solve optimization problems over time [19,20]. They are effective in materials design as they can explore numerous potential solutions quickly. Combined with physical models at the atomic or molecular level, GAs optimize coating composition and heat treatment conditions [21,22]. Moreover, support vector regression (SVR), an ML model based on statistical learning theory, predicts material properties accurately. SVR is suitable for predicting continuous outcomes like hardness or wear resistance of multi-metal nitride coatings [23,24,25,26,27,28]. Its strength is finding the optimal hyperplane for data fitting, making it great for property prediction in complex systems.

Combining GAs and SVR enables researchers to predict the performance of ternary metal nitrides in multi-metal systems. This combined method allows for rapid screening of potential material configurations, speeding up discovery. The GAs with an elite strategy retain top-performing solutions during optimization, and the SVR model improves property prediction accuracy by using intermediate variables from physical theories, refining data for better exploration of the compositional space.

Nonetheless, challenges and controversies exist in multi-metal nitride hard coating design. A key problem is the dataset size and quality for ML models. ML models like SVR perform best with large and high-quality data. However, in multi-metal nitride design, data are often scarce due to experiment costs, time, or data privacy. Small dataset models may overfit and struggle to generalize.

Moreover, integrating physical models into ML frameworks shows potential but is debated. Incorporating theoretical insights as intermediate variables is a controversial challenge. Some researchers argue that existing theories, such as those describing the relationships between composition, microstructure, and properties, are not fully utilized in current ML frameworks [25,26]. They contend that by more deeply embedding these theories into ML models, the predictions could be made more reliable and interpretable. However, doing so introduces additional complexity, making the models harder to train and increasing the computational resources required.

Another controversial area is the variability of experimental conditions. The performance of multi-metal nitride coatings is very sensitive to synthesis and testing conditions like temperature, pressure, and deposition time. Small parameter variations can cause significant property differences, making modeling and prediction difficult. Some researchers doubt if current ML models can capture this variability and wonder if new approaches are needed to consider the complex interactions between composition and processing conditions. To tackle these challenges, our study introduces a GA-SVR methodology that enhances prediction accuracy by integrating physical models and addressing data limitations. To address challenges of small datasets, integrating physical models with ML, and experimental condition variability, the GA-SVR methodology in this study is a significant advancement in multi-metal nitride coating design. By using existing theories as intermediate variables, it enhances data quality and prediction accuracy, enabling more efficient exploration of complex compositional systems, which is crucial in multi-metal systems where traditional experiments are time-consuming and costly.

This approach not only shows potential for better prediction accuracy but also can shorten the development cycle. In industry, quickly identifying promising candidates and validating performance is a major plus. The methodology offers a valuable framework for future R&D in material design.

This study begins by establishing a search space based on the micro- and mesoscopic properties of unary nitride hardness (Section 2.1), followed by feature engineering using solid solution strengthening theory (Section 2.2) and the development of the GA-SVR model (Section 2.4). Building on this foundation, the prediction model is constructed (Section 3), starting with the GA’s fitness function, chromosome coding for alloy compositions (e.g., molar ratios of metal elements), and population initialization (Section 3.1), linking computational optimization to alloy design. Section 4 (Results) presents the model’s predictions, and Section 5 (Prediction Results and Experimental Validation) validates these predictions against experimental hardness measurements.

In conclusion, this study aimed to develop an efficient and accurate machine learning framework combining a genetic algorithm (GA) and support vector regression (SVR) to design high-hardness multi-metal nitride coatings, addressing the inefficiencies of traditional trial-and-error methods. It has advantages over traditional methods, reducing discovery time and cost and enabling more accurate property predictions. The study’s insights benefit the multi-metal nitride coating field and offer a broader basis for future material design innovations.

The need for additional research arose from the limitations of traditional trial-and-error methods, which are inefficient and costly for designing multi-metal nitride coatings due to the vast compositional search space and sensitivity to experimental conditions such as temperature and pressure. Prior studies often utilized idealized models that failed to integrate micro-, meso-, and macro-scale properties or account for experimental variability, resulting in suboptimal predictive accuracy. Furthermore, the small dataset size (233 entries, Section 2.1) compared to large datasets (tens of thousands to millions) or medium datasets (hundreds to thousands) in other machine learning applications posed a unique challenge. This study addresses these gaps by developing a GA-SVR framework that leverages advanced feature engineering and optimization to enhance predictive accuracy and efficiency, enabling rapid exploration of complex multicomponent nitride systems.

## 2. Problem Description of Hardness Prediction Model

### 2.1. Establishment of Searching Space

**Figure 1:**
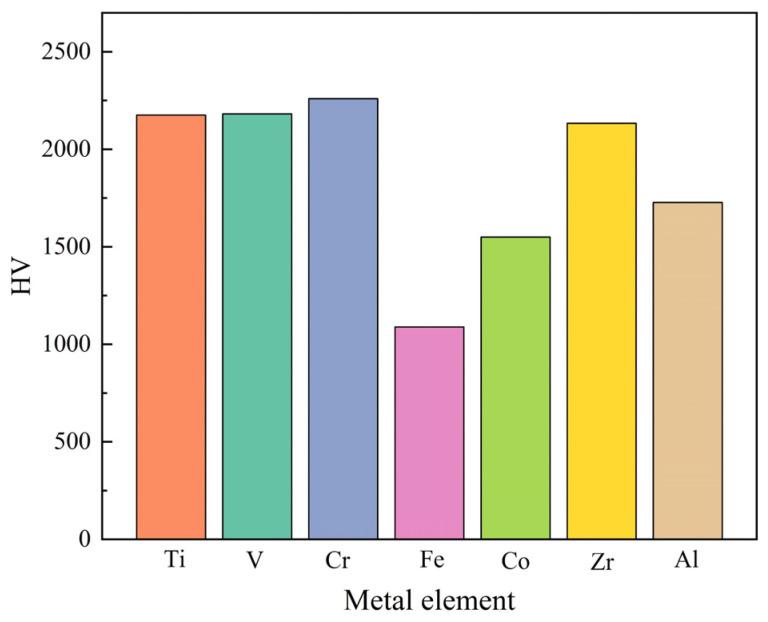
Hardness of single metal nitrides.

To build the search space for metal elements, this study identified high-hardness unary metal nitrides via a comprehensive literature survey. Figure 1 illustrates a high-hardness unary nitride (e.g., TiN), highlighting its key properties such as valence electron count and electronegativity, which guide the selection of metal elements (e.g., Ti, Al, Cr, Zr) for constructing the multicomponent nitride search space in the GA-SVR model. The selected metal elements form the set ME (Equation (1)), which is the search space for nitrides in this study. In the nitrides system, the micro-mechanisms of unary nitride hardness, including chemical bonding and electronic structures, provide a fundamental theoretical basis for understanding multicomponent nitride performance [29,30,31,32,33,34]. These mechanisms offer essential insights for analyzing the complex interactions determining multicomponent nitride properties.

An analysis of the seven metal elements in ME reveals shared characteristics, including outermost electron orbitals of 4s, 3d, or 4d; electronegativity values ranging from 1.54 to 1.91; and common oxidation states of +1, +2, +3, +4, +5, and +6. These similarities in electronic configuration, valence electron count, electronegativity, and oxidation states significantly influence the physical and chemical properties of metal nitrides, such as hardness, stability, and reactivity [32,33]. Based on these findings, the set MP (Equation (2)), comprising these four attributes, is incorporated into the model’s search space and utilized as critical features in the model.

(1) ME={Ti,V,Cr,Fe,Co,Zr,Al}

(2) $$MP=\{(E_{i},V_{i},X_{i},O_{i})|i\in M\}$$

Here, *E_i_* represents the electronic configuration, *V_i_* the valence electron count, *X_i_* the electronegativity, and *O_i_* the oxidation state of each metal element in *M*. The mapping function *f* establishes the relationship from *MP* to *ME* (Equation (3)).

(3) f:ME→MP

This study focuses on the strengthening mechanisms of multicomponent FCC-structured nitrides under specific experimental conditions and datasets [35,36]. A subset of *ME*, denoted as ME_Sub (Equation (4)), is derived to define the search space for metal elements in the model, with its relationship to *ME* expressed as Equation (5).

(4) ME_Sub={Cr, Ti, Al, Zr}

(5) ME_Sub⊆ME

To support the research, a dataset was compiled from the literature, encompassing FCC metal nitrides prepared via the multi-arc ion plating method. This dataset contains 233 entries, including 4 unary metal nitrides, 34 binary metal nitrides, and 195 ternary metal nitrides. These data provide a robust experimental foundation and theoretical framework for the performance prediction and optimization of multicomponent nitrides.

### 2.2. Problem Description and Model Assumptions

According to the “No Free Lunch Theorem”, it is challenging to identify an optimal ML model applicable to all materials problems [37]. To enhance the predictive accuracy of the model, we constructed a feature space based on solid solution strengthening theory [38,39]. Thus, incorporating the solid solution strengthening mechanism into the SVR model, along with relevant constraints added through the GA as the top-level optimization algorithm, is critical for improving predictive performance.

To establish these constraints, the following derivations are made. Assuming the metal nitride is an ideal solid solution and all crystal structures in the selected dataset are FCC, the predictions are based on this FCC structure. The derivation proceeds as follows:

Case 1: Ternary Metal Nitride as Ideal Substitutional Solid Solutions

In an ideal substitutional solid solution, the solute atoms replace solvent atoms in a 1:1 ratio, leading to a constrained relationship between metal atoms and N atoms [40]. Based on this assumption, four states with varying solute concentrations are discussed:

Low Solute Concentration: On the condition of low solute concentrations, solute atoms randomly substitute solvent atoms, forming a disordered solid solution. In this state, solvent atoms still dominate the lattice structure, and the ratio of solute to solvent atoms does not induce significant lattice changes [41].

Moderate Solute Concentration: As the solute concentration increases, solute atoms start to significantly affect lattice properties, such as lattice constants, electronic structure, and material properties. Although the contribution of solute atoms increases, the lattice structure remains primarily controlled by the solvent atoms, and solute atoms have not yet altered the dominant characteristics of the lattice [39,40]. The relationship between metal atoms and N atoms at low and moderate solute concentrations can be expressed as Equation (6):

(6) $$\frac{\left(a_{1}+b_{1}\right)}{c_{1}}=\frac{\left(a_{2}+b_{2}\right)}{c_{2}}$$

where *a_n_* represents the moles of solvent atoms, *b_n_* represents the moles of solute atoms, and *c_n_* represents the moles of N atoms, with n denoting the current state.

Exceeding Solid Solution Limit: When the solute concentration reaches the critical point (the solid solution limit), the lattice structure undergoes significant changes. At this stage, the interaction between solute and solvent atoms becomes more balanced, with the solute atoms sometimes dominating certain physical properties, forming a solute–solvent synergistic effect [41,42]. When the solute concentration exceeds this limit, the lattice structure changes, and solute atoms take over the lattice.

High Solute Concentration: At very high solute concentrations, solute atoms dominate the lattice structure, surpassing the solvent atoms, which are now effectively “dilute solutes”. This typically occurs in alloy systems where the solute and solvent have highly similar physical and chemical properties [41].

In the third and fourth cases, the interaction between metal and N atoms shifts from weak to stronger associations, signifying a structural change in the positions and roles of solute and solvent atoms in the lattice. In this context, the relationship between metal atoms and N atoms is expressed as Equation (7):

(7) $$\frac{\left(a_{3}+b_{3}\right)}{c_{3}}=\frac{\left(a_{4}+b_{4}\right)}{c_{4}}\neq \frac{\left(a_{1}+b_{1}\right)}{c_{1}}$$

Case 2: Ternary Metal Nitride as Complete Interstitial Solid Solutions

In contrast to substitutional solid solutions, in complete interstitial solid solutions, the solute atoms occupy interstitial positions in the solvent crystal lattice, rather than directly replacing the solvent atoms. Due to their smaller size, interstitial atoms can be accommodated in the lattice voids, giving these solutions a solubility limit [41]. Based on this characteristic, the relationship between metal atoms and N atoms in ternary metal nitride is expressed as Equation (8):

(8) $$\frac{a}{c}<\frac{\left(a+b\right)}{c}\leq \frac{(1+\delta_{\max})a}{c}$$

where *a*, *b*, and *c* represent the moles of solvent atoms, solute atoms, and N atoms, respectively, and *δ*_max_ is a constant taken as 0.022 based on the solubility limits of typical interstitial solid solutions [43,44].

Case 3: Mixed Solid Solutions

In mixed solid solutions, both substitutional and interstitial solid solutions coexist. In this case, the relationship between metal atoms and N atoms in ternary metal nitride is similar to that in Case 2 and is expressed by Equation (8), as no further distinction is necessary.

### 2.3. The Establishment of Model Constraints

The proposed model, based on the search space defined in Section 2.1, aims to predict the hardness of ternary metal nitrides. As analyzed in Section 2.2, the atomic composition of ternary metal nitrides is closely related to the corresponding unary nitrides. Therefore, we first selected unary nitrides with FCC lattices for Ti, Al, Cr, and Zr from the Materials Project (https://materialsproject.org/, accessed on 4 June 2025), forming a subset labeled “subset-N” (see Supplementary Table S1 for details).

Building on the results from Section 2.2, the model’s constraints are defined. The molar ratios of the elements are used to represent their proportions in the compound, denoted as *x*_1_, *x*_2_, *x*_3_, *x*_4_, and *x*_5_ for Ti, Al, Cr, Zr, and N, respectively. To ensure the formation of nitrides, the molar ratio of N element (*x*_5_) must be nonzero. Following the above analysis 1, we formulate Constraint 1 and Constraint 2, which are expressed as follows:

(9) $$\mathrm{Constraint}\;1:\;\sum_{i=1}^{n}x_{i}=1\;\;\left(\mathrm{if}\;\;\mathrm{and}\;\;\mathrm{only}\;\;\mathrm{if}\;x_{i}=0,\;(i=1,2,3,4\right)$$

(10) $$\mathrm{Constraint}\;2:\;\eta_{ratio}<\frac{\sum_{i=1}^{4}x_{i}}{x_{5}}\leq \eta_{ratio}+\delta_{\max}\;\;\;\;\;\;(\eta_{ratio}\in subset-N)$$

### 2.4. Selection of Model Features and Establishment of Objective Function

Unlike traditional ML methods, which typically rely on raw inputs such as composition and process parameters to directly associate with target output properties [45,46], this study introduces a feature library that systematically incorporates micro-, meso-, and macro-structural characteristics of materials. These structural features play a crucial role in bridging the raw inputs and target outputs and must be considered comprehensively [47,48,49].

In principle, the more comprehensive the features included in the model, the more complete the search space, which in turn improves prediction accuracy and coverage [50]. Therefore, this study broadens the parameter space by collecting as many features as possible and converts these features into descriptors recognizable by ML algorithms, enhancing the model’s predictive capability. Based on elemental parameters, we developed two statistical models (Equations (11) and (12)) to calculate statistical data for multicomponent compounds. For subsequent analysis, all features were grouped into four subsets (Supplementary Table S2).

(11) $$\overset{-}{x}={\sum c_{i}x}_{i}$$

(12) $$\delta_{x}=\sqrt{\sum c_{i}(1-x_{i}/\overset{-}{x})}$$

where *c_i_* represents the molar percentage of atoms and *x_i_* is the characteristic of component *I*; if $\overset{-}{x}$ is zero, then $x_{i}/\overset{-}{x}$ is also zero.

Once the dataset and feature sets are constructed, an SVR model can be established. SVR, a regression method based on statistical learning theory, fits data by constructing a hyperplane with a maximum margin, ensuring that the distance from all training samples to the hyperplane is within a given ε [0.05, 0.2] range, expressed as follows (Equation (13)).

(13) $$K(x_{i},x_{j})=\exp(-\frac{{\left|x_{i}-x_{j}\right|}^{2}}{2\sigma^{2}})$$

where σ is the kernel parameter that controls the width of the Gaussian distribution. The normalization condition is applied to the input features x_i_ and x_j_, which are scaled to [0,1] to ensure consistent kernel behavior across differing feature scales (e.g., molar ratios, valence electron count). However, K(x_i_, x_j_) itself is not normalized, as its output is inherently bounded by the RBF kernel and controlled by σ, avoiding redundancy and preserving the intended mapping.

In most ML models, the objective function is typically a clear mathematical expression designed to directly optimize a specific performance metric. However, in this study, the objective function employs the SVR method [51,52]. This design contrasts with traditional explicit objective functions, as SVR establishes a data-driven nonlinear regression model to achieve optimization. By using SVR as the objective function, the model performs nonlinear mapping in a high-dimensional feature space, allowing it to more flexibly adapt to the complex relationship between input features and target outputs [53]. The advantage of this approach is that SVR not only captures the nonlinear characteristics of the data but also enhances the model’s generalization ability by maximizing the margin, thus achieving an effective balance between prediction accuracy and model robustness.

## 3. The Establishment of an Intensity Prediction Model Algorithm

**Figure 2:**
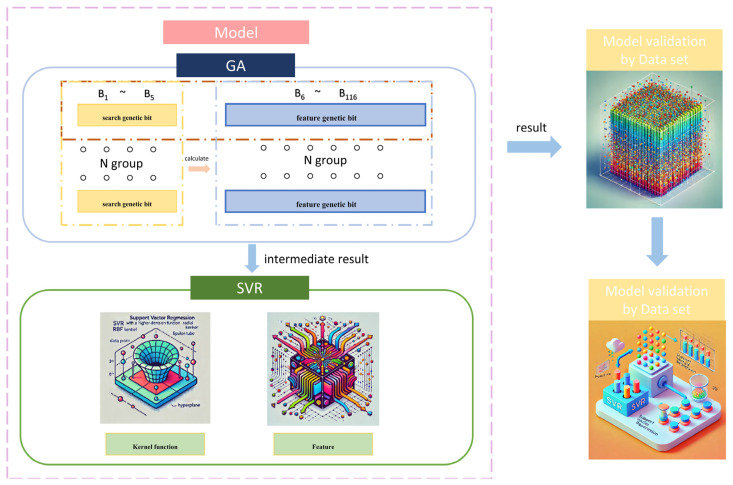
Schematic diagram of hardness prediction model algorithm.

The model presented in this study integrates two algorithms: GA with an elitist strategy and SVR. The GA, with its robust global search capability, effectively avoids local optima in complex solution spaces, offering broad exploration during the optimization process. In contrast, SVR, a regression technique that partitions data to search for a hyperplane, features a simple structure and demonstrates high computational efficiency, particularly on small datasets, allowing for rapid model training and prediction. The combination of these two algorithms leverages the GA’s global search advantages and the SVR’s efficiency with small datasets, thereby enhancing both the prediction accuracy and computational efficiency of the model. Figure 2 illustrates the architecture of the GA-SVR model, where the GA (top) optimizes the feature set, including molar ratios of Ti, Al, Cr, Zr, and derived structural characteristics (e.g., electronic configuration, valence electron count), and passes these to the SVR (bottom) for hardness prediction. The figure also shows the training process, where the dataset of 233 entries is used to train the SVR model, with the GA iteratively refining inputs to minimize prediction errors.

### 3.1. Fitness Function of Strength Prediction Model, Chromosome Coding, and Population Initialization

This study presents a model built upon GA and SVR. A fitness function is constructed to assess the quality of candidate solutions, with the primary objective of predicting the hardness of ternary metal nitrides. In this model, the output of the objective function represents the required hardness prediction, thereby linking the objective function with the fitness function and ensuring functional consistency, as exemplified in the SVR model developed in Section 2.3.

The direct relationship between the objective and fitness functions ensures that the genetic algorithm effectively explores the solution space. Through iterative optimization, it progressively enhances the quality of candidate solutions, thereby achieving the set optimization goal [51,52]. This design not only streamlines the algorithm’s implementation, focusing on the core problem of hardness prediction, but also enhances the algorithm’s transparency and interpretability, providing a solid theoretical foundation for the reliability of the research outcomes.

As discussed in Section 2.2, the type of solid solution influences the associated constraints, necessitating the identification of the solid solution type before encoding. The formation of solid solutions is governed by five key factors: atomic size similarity, chemical affinity, valence electron concentration, lattice type compatibility, and thermodynamic conditions. In this study, atomic size similarity is used as the primary criterion for determining the solid solution type, as described in Equation (14):

(14) $$\left|r_{solute}-r_{solvent}\right|\leq \Delta r_{max}$$

Here, *r_solute_* represents the atomic radius of the solute; *r_solvent_* represents the atomic radius of the solvent; and ∆*r_max_* represents the maximum permissible atomic radius difference, typically set to 0.15.

Based on the atomic size similarity criterion, a solid solution determination coefficient is constructed as follows:

(15) $$\delta_{ss}=\{\begin{array}{c} 0\;\;\;\;\;\;\;\;\;\;\;all\;\;\;\left|r_{i}-r_{j}\right|\leq \Delta r_{\max}\;\;\;\;\;\;i,j\in M\\ \delta_{\max}\;\;\;\;\;\;else \end{array}$$

where *M* represents the set of atomic radii for the metal elements involved in the model, specifically Cr, Ti, Al, and Zr.

(16) $$\begin{array}{l} f(B_{k1})=0\\ f(\eta_{ration},\delta_{ss})=\frac{\eta_{ration}}{1+\eta_{ration}}\begin{array}{c} +\theta (\frac{\eta_{ration}+\delta_{ss}}{1+\eta_{ration}+\delta_{ss}}-\frac{\eta_{ration}}{1+\eta_{ration}}) \end{array}\\ f(B_{k2})=\lambda_{1}f(\eta_{ration},\delta_{ss})\\ f(B_{k3})=\lambda_{2}f(\eta_{ration},\delta_{ss})\\ f(B_{k4})=\lambda_{3}f(\eta_{ration},\delta_{ss})\\ f(B_{5})=1-f(\eta_{ration},\delta_{ss})\\ k_{i}\in \{1,2,3,4\}\;\;\;k_{i}\neq k_{j}\;\;\;i,j=1,2,3,4\;\;\;\lambda_{1}+\lambda_{2}+\lambda_{3}=1\;\;\;\;\;\;0<\theta \leq 1 \end{array}$$

In general, population initialization in GA is achieved by random generation of individuals. However, due to the need to satisfy constraints 1 and 2 in the model, and to minimize the waste code rate, specific initialization methods are applied to the first five positions of the chromosome. Specifically, these positions are initialized based on Equation (16), ensuring that the genes of each individual conform to the solid solution type criteria.

**Figure 3:**
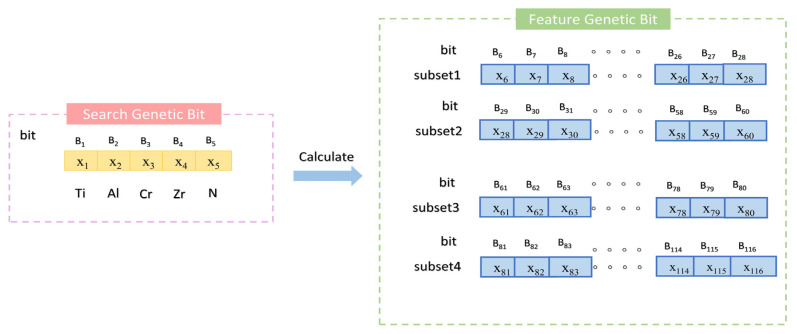
Schematic diagram of population initialization in algorithm.

The hardness calculation model consists of two main components (Figure 3); the first involves searching within the molar ratio space of Ti, Al, Cr, Zr, and N elements in the ternary metal nitride system, and the second involves computing the features of the identified compounds to evaluate their fitness. Therefore, the chromosome structure is divided into two parts. The first part, from the first to the fifth gene positions, corresponds to the molar ratios of the aforementioned elements, termed the “search gene positions”. The second part, from the 6th to the 45th gene positions, calculates the relevant features of the nitrides identified in the search, using Equation (11), while the 46th to the 85th positions are derived using Equation (12). Notably, all gene positions in the chromosome are encoded in decimal format.

In summary, the population initialization method described above generates the chromosomes for each individual in the population. After initialization, each chromosome represents an initial solution to the model, forming a population of 100 chromosomes that serves as the foundation for the subsequent genetic algorithm optimization process.

### 3.2. Chromosome Roulette Selection and Crossover Operator Design

The selection of chromosomes within the population is a crucial step in the evolutionary process of GA. In this model, a roulette wheel selection strategy is employed to select the chromosomes for the next generation according to Equation (17).

(17) $$P_{i}=\frac{F_{i}}{\sum_{i=1}^{N}F_{i}}\;\;\;\;\;\;i\in N$$

(18) $${P_{i}}^{sum}=\sum_{k=i}^{i}P_{k}\;\;\;\;\;\;i\in N$$

**Figure 4:**
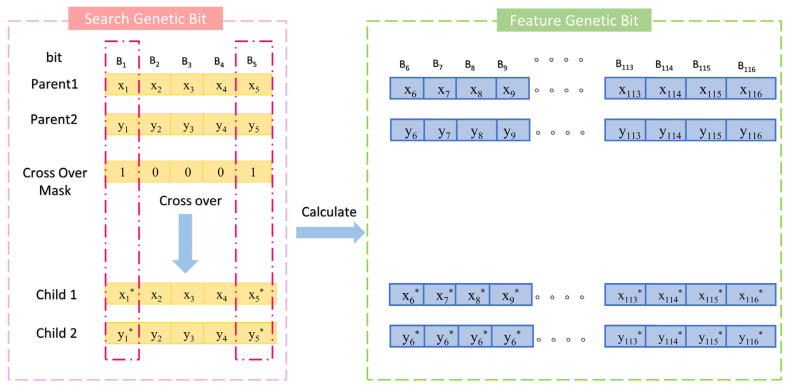
Schematic diagram of crossover strategy in algorithm. A crossover mask value of 1 produces new encodings through crossover, while a value of 0 preserves the original encoding. Thus, the asterisk (*) symbol marks positions where new encodings are generated.

Traditional crossover operators, such as single-point or uniform crossover, are not directly applicable in this model due to the unique requirements of decimal encoding and the specific stoichiometric and physical constraints of multi-metal nitride compositions. Therefore, an innovative crossover algorithm suitable for decimal encoding and model constraints is proposed (Equation (19)). This algorithm combines the advantages of multi-point and arithmetic crossover, leveraging the flexibility of multi-point crossover to select multiple gene segments and the precision of arithmetic crossover to blend numerical values, thus preserving the continuity of decimal-encoded molar ratios. The algorithm is implemented in two stages: First, a genetic crossover stage, where the crossover is carried out based on a five-bit crossover mask. The genes corresponding to the mask value of 1 are crossed using the arithmetic crossover formula. Second, a gene repair stage (Equation (20)) ensures that the newly generated genes conform to the model’s constraints, such as maintaining the sum of molar ratios to 1 and ensuring non-negative values, by normalizing or adjusting the genes post-crossover (Section 3.1). This repair mechanism is critical to producing valid offspring that align with the physical and chemical properties of nitride coatings (Figure 4).

(19) $$\begin{array}{l} f({C^{1}}_{B_{i}})=(f({P^{1}}_{B_{i}})+\lambda (f({P^{1}}_{B_{i}})-f({P^{2}}_{B_{i}}))\\ f({C^{2}}_{B_{i}})=(f({P^{2}}_{B_{i}})+\lambda (f({P^{2}}_{B_{i}})-f({P^{1}}_{B_{i}})) \end{array}$$

(20) $$\begin{array}{l} \eta_{\mathrm{ratio}-C^{m}}=\eta_{\mathrm{ratio}-p^{m}}\;\;\;\;\;\;\;\;\;m=1,2\\ f(B_{k1-C^{m}})=0\\ f(\eta_{ration-C^{m}},\delta_{ss})=\frac{\eta_{ration-C^{m}}}{1+\eta_{ration-C^{m}}}\begin{array}{c} +\theta^{\prime }(\frac{\eta_{ration-C^{m}}+\delta_{ss}}{1+\eta_{ration-C^{m}}+\delta_{ss}}-\frac{\eta_{ration-C^{m}}}{1+\eta_{ration-C^{m}}}) \end{array}\\ f(B_{k2-C^{m}})={\lambda^{\prime }}_{1}f(\eta_{ration-C^{m}},\delta_{ss})\\ f(B_{k3-C^{m}})={\lambda^{\prime }}_{2}f(\eta_{ration-C^{m}},\delta_{ss})\\ f(B_{k4-C^{m}})={\lambda^{\prime }}_{3}f(\eta_{ration-C^{m}},\delta_{ss})\\ f(B_{5-C^{m}})=f(B_{5-P^{m}})\\ k_{i-C^{m}}\in \{1,2,3,4\}\;\;\;k_{i-C^{m}}\neq k_{j-C^{m}}\;\;\;i,j=1,2,3,4\;\;\;{\lambda^{\prime }}_{1}+{\lambda^{\prime }}_{2}+{\lambda^{\prime }}_{3}=1\;\;\;\;\;\;\;\;\;0<\theta^{\prime }\leq 1 \end{array}$$

### 3.3. Variation Operator Design and Elite Strategy

**Figure 5:**
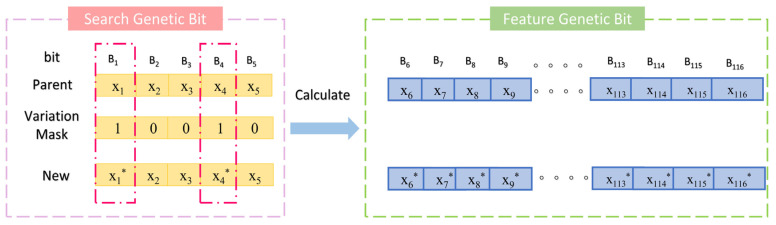
Schematic diagram of variation strategy in algorithm. A mutation mask value of 1 introduces new encodings through mutation, while a value of 0 preserves the original encoding. The asterisk (*) explicitly marks positions where mutations occur.

To enhance the stability and robustness of the algorithm, a mutation mechanism is incorporated, comprising two components (Figure 5): the design of a mutation mask and the definition of a mutation probability. Each gene position on the chromosome is assigned an independent mutation probability (Equation (21)). Following mutation, all chromosomes are evaluated against Constraint 1 and Constraint 2 to ensure validity. Any chromosome failing to meet these constraints is repaired using Equation (20).

(21) $$\begin{array}{l} f({B^{*}}_{B_{i}})=f(B_{B_{i}})+\Delta f(B_{B_{i}})=0\;\;\;\;\;\;(i=1,2,\ldots \ldots ,n)\\ \Delta f(B_{B_{i}})=\theta f(B_{B_{i}})\left(-0.3\leq \theta \leq 0.3\right) \end{array}$$

In the GA, interdependencies among gene positions within an individual are common. Simple and independent application of crossover and mutation operations to each gene position can disrupt beneficial gene combinations, hindering the accumulation of high-quality genetic information. To address this issue, an elite strategy is incorporated, which preserves the genetic information of the best-performing individuals to enhance convergence and global optimization.

**Figure 6:**
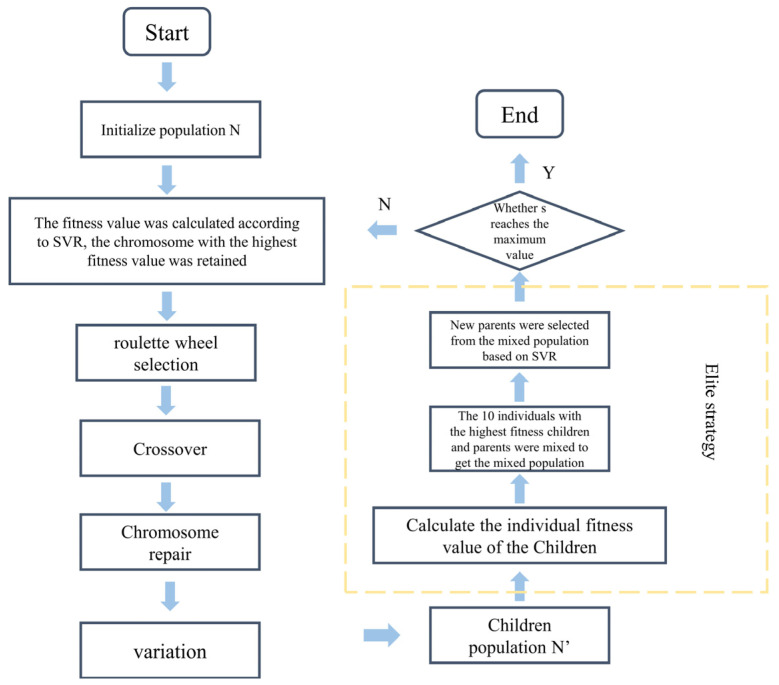
Diagram of the Elite Strategy.

The implementation of the elite strategy involves the following steps (Figure 6):

1. Fitness Evaluation: Evaluate the fitness of all individuals in the population and identify the highest-performing individuals.
2. Elite Retention: Directly copy the top 10 individuals with the highest fitness scores to the next generation.
3. Standard Genetic Operations: Apply conventional genetic operations, such as crossover and mutation, to the remaining individuals to generate new candidate solutions.
4. Generation of the New Population: Combine the retained elite individuals with the newly generated candidates to form the next generation.

Additionally, a clear stopping criterion is established for the genetic algorithm: the search terminates when the number of iterations reaches 800 generations, at which point the best solution in the current population is output as the final result. By preserving optimal solutions, the elite strategy accelerates convergence and improves global optimization performance, ensuring the algorithm remains robust and efficient when addressing complex optimization problems.

## 4. Experimental Methods

### 4.1. Hardness Precipitate Details

This study compiles compositional and hardness data for various ternary metal nitrides reported in the literature, all of which exhibit a face-centered cubic (FCC) crystal structure. To construct the predictive model, the elemental ratios of compounds in a selected dataset were used as the initial input variables. For model optimization, the algorithm parameters were set as follows: a population size of 400, a crossover rate of 0.85, a mutation rate of 0.0035, and 800 iterations. To comprehensively evaluate the model’s performance, additional independent datasets were employed for validation. Multiple predictions were performed for each sample to assess the model’s generalization capability, ensuring robustness and applicability across different datasets.

### 4.2. SVR Model Accuracy Detection

To evaluate the generalization ability of the model, the collected dataset was processed as follows. Given the limited size of the dataset (only 233 samples), the way the training and testing sets are split has a significant impact on model performance. Additionally, random data splitting can lead to imbalanced distributions, introducing potential biases in the evaluation. To provide a more comprehensive assessment, this study employed k-fold cross-validation. Specifically, the 233 samples were randomly divided into 10 groups, each containing approximately 70 samples, ensuring that each sample participated in both training and testing multiple times. In each iteration, 9 groups were randomly selected as the training set, while the remaining 1 group served as the test set. This process was repeated 10 times, ensuring that each group was used as a test set at least once.

(22) $$MAE=\frac{1}{n}\sum \left(x_{i}-y_{i}\right)$$

(23) $$MSE=\frac{1}{n}{\sum \left(x_{i}-y_{i}\right)}^{2}$$

where *n* represents the number of samples, and *x_i_* and *y_i_* denote the predicted and actual values for the *i*th sample, respectively.

(24) $$SST={\sum \left(y_{i}-\frac{1}{n}\sum y_{i}\right)}^{2}$$

(25) $$SSR={\sum \left(x_{i}-\frac{1}{n}\sum y_{i}\right)}^{2}$$

(26) $$R^{2}=\frac{SSR}{SST}$$

To quantify the model’s generalization capability, we evaluated its performance using metrics including Mean Absolute Error (MAE) and Mean Squared Error (MSE), with calculation formulas defined in Equations (22) and (23). Additionally, the coefficient of determination (R^2^) was calculated using the regression sum of squares (SST) and total sum of squares (SSR) to measure the model’s ability to explain the variance in the data. The relevant formulas are provided in Equations (24)–(26). Here, SST represents the variance explained by the model, and SSR represents the total variance in the data. The value of R^2^ ranges from 0 to 1; the closer the value is to 1, the better the model’s explanatory power and fit to the data. Conversely, an R^2^ value near 0 indicates a weak explanatory power and poor model fit.

### 4.3. Materials and Multi-Arc Ion Experiment 

M2 high-speed steel substrate (Φ40 mm × 5 mm, pre-treated, Huangshi) was purchased from Daye Special Steel Co., Ltd., Huangshi, Hubei, China. High-purity argon gas (Ar, 99.999%) and nitrogen gas (N_2_, 99.999%) were obtained from Shanghai Chinllenge Gases Co., Ltd., Shanghai, China, and Wuhan Newradar Special Gas Co., Ltd., Wuhan, Hubei, China, respectively. Chromium-Titanium (Cr-Ti) alloy target material (99.99% purity) and Aluminum-Zirconium (Al-Zr) alloy target material (99.99% purity) for plasma coating were sourced from Beijing Zhongnuo Advanced Material Technology Co., Ltd., Beijing, China. 

The pretreated Φ40 mm × 5 mm M2 high-speed steel substrate was placed on a rotating holder in a vacuum chamber. The chamber was evacuated, and the baking current was gradually raised to 90 A for proper deposition temperature. When the substrate reached 120 °C and the chamber pressure dropped below 2.0 × 10^−2^ Pa, Ar was introduced at a flow rate of 12–13 sccm to initiate plasma cleaning, ensuring a clean substrate surface for subsequent coating deposition.

Metallic transition layer deposition followed to strengthen the coating–substrate bond for 5 min. Then, N_2_ was introduced as a reactive gas. The N_2_ flow rate was dynamically adjusted based on real-time pressure monitoring data to maintain a stable partial pressure of 0.38 Pa throughout the heating process as the furnace temperature was gradually increased from ambient to 800 °C at a controlled rate of 5 °C/min. The 15-min deposition process used a constant bias voltage of 180 V for a uniform and high-quality film.

### 4.4. Test Equipment

The surface and cross-sectional morphologies of the coatings were characterized using a ZEISS field emission scanning electron microscope (SEM) (Carl Zeiss Microscopy GmbH, Oberkochen, Germany) to analyze their microstructural features. The three-dimensional surface morphology and roughness of the coating were measured using a TaylorSure CCl2000 (Taylor Hobson Ltd., Leicester, UK) non-contact surface profilometer, providing a comprehensive assessment of the surface’s microscopic geometry. The phase composition of the coating was analyzed using an XRD-7000 X-ray diffractometer (Shimadzu Corporation, Kyoto, Japan), with a monochromatic Al Kα (150 W, 15 kV, 1486.71 eV).

The critical load for coating adhesion was determined using a WS-2005 scratch adhesion tester (Zhuhai Lisite Science & Technology Co., Ltd., Zhuhai, China), with testing conditions including a preload of 0.1 N, a sliding speed of 5 mm/min, and a loading rate of 60 N/min. Each sample surface was tested three times, and the average value was recorded to ensure result reliability.

## 5. Discussion

### 5.1. Feature Set Analysis

The algorithm proposed in this study demonstrates significant distinctions from traditional modeling approaches. Conventional models typically rely solely on raw input data, such as composition and processing parameters, as input variables. However, in materials science, microstructural and mesoscale features, along with thermodynamic and kinetic properties, play critical roles in bridging the gap between raw inputs and target output properties [53,54,55,56]. To address this, this study employs an improved GA to optimize within the feature space, refining the molar ratios of elements in ternary metal nitrides. Based on the optimization results and selected features, SVR is utilized as the objective function to establish a quantitative relationship between elemental molar ratios and material hardness.

In this study, SVR is employed not only as the core objective function of the model but also to evaluate its generalization capabilities. To achieve this, cross-validation methods were applied using the dataset, and feature evaluations were conducted by calculating AUC (Area Under the Curve) values for all features listed in Table 1. AUC is a widely used metric for assessing the performance of binary classification models and is derived from the ROC (Receiver Operating Characteristic) curve. The ROC curve illustrates the trade-off between the True Positive Rate (TPR) and False Positive Rate (FPR) at various decision thresholds, providing an intuitive representation of how the model’s classification performance changes with varying thresholds. In this context, higher-quality features result in ROC curves closer to the upper-left corner of the plot, indicating higher TPRs and lower FPRs.

The AUC value, representing the area under the ROC curve, ranges from 0 to 1 and quantifies the classification performance of the model. It can be interpreted as the probability that a randomly selected positive sample is ranked higher than a randomly selected negative sample by the model. An AUC value closer to 1 indicates superior classification performance, with the model accurately distinguishing between positive and negative samples. Conversely, an AUC value near 0.5 suggests performance akin to random guessing, reflecting poor classification ability. Through this evaluation method, the study not only validates the fitting capability of the SVR model but also comprehensively assesses its generalization performance in practical applications, providing robust quantitative support for further optimization.

(27) $$TPR=\frac{TP}{TP+FN}$$

In this context, TP (True Positive) refers to the number of true positives, while FN (False Negative) refers to the number of false negatives.

(28) $$FPR=\frac{FP}{FP+FN}$$

In this context, FP (False Positive) refers to the number of false positives, while TN (True Negative) refers to the number of true negatives.

**Figure 7:**
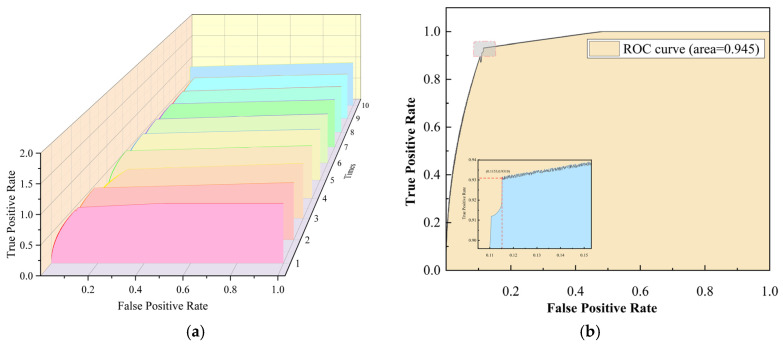
(**a**) Detection performance with all features by ten times. (**b**) The first-time detection performance with all features.

In this study, the proposed model underwent a comprehensive performance evaluation using the aforementioned methodology, with its classification capability visualized through 10 sets of ROC curves (Figure 7). ROC curves closer to the upper-left corner of the plot indicate superior performance in distinguishing between positive and negative samples. By analyzing these curves, the model’s classification effectiveness and predictive capability across varying decision thresholds were intuitively assessed.

In the initial test, the model demonstrated high regression accuracy. Notably, by examining the inflection point of the ROC curve, the accuracy within the top 10% prediction range was estimated to be 90%. This result was further corroborated by the AUC values, underscoring the model’s exceptional classification performance within this range. Specifically, the AUC quantifies the model’s classification ability across different thresholds, with values closer to 1 indicating superior performance.

As summarized in Table 1, the AUC values from 10-fold cross-validation varied across experiments but yielded an average of 0.941. This average reflects a high degree of consistency in the model’s classification performance across multiple tests, along with robust generalization capability. Overall, the near-unity AUC value further validates the model’s high predictive accuracy and reliability in practical applications, highlighting its strong classification proficiency and remarkable stability.

This study employs a linear support vector machine (SVM) coefficient analysis method to quantitatively evaluate the importance of features within the dataset. To facilitate comparative analysis, each feature’s importance coefficient was normalized to a relative value between 0 and 1, with the sum of all feature importance coefficients constrained to equal 1. A higher coefficient value indicates a more significant contribution of the feature to the model’s predictive performance, reflecting its relative importance in the feature set.

**Figure 8:**
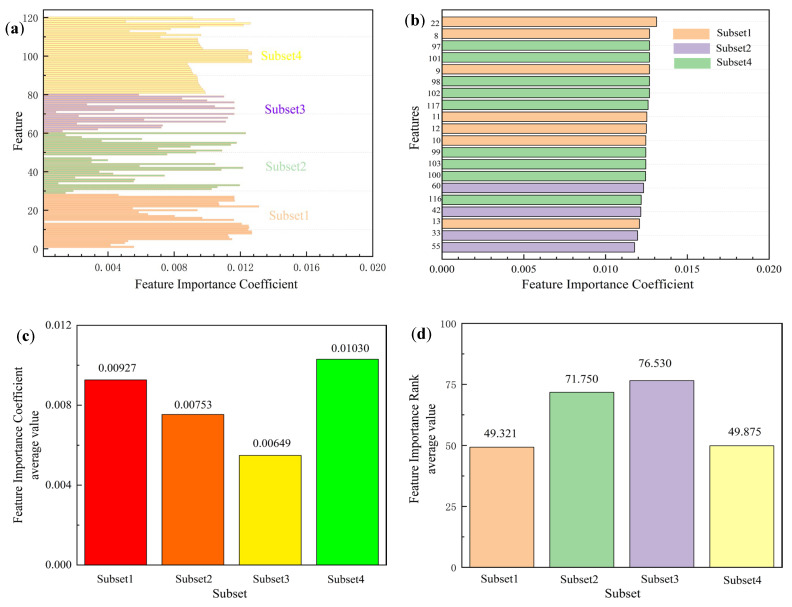
(**a**) The feature importance coefficient of all sets. (**b**) The feature importance coefficient of the top 20 in all sets. (**c**) The average value of the feature importance coefficient of all sets. (**d**) The average value of the feature importance coefficient rank of all sets.

As shown in Figure 8a, the analysis of feature importance coefficients reveals that subset 1 contains the highest number of features but has a lower average importance coefficient compared to subset 4. In contrast, although subset 4 comprises fewer features, its average importance coefficient is the highest. This finding indicates that the contributions of features within subset 1 are relatively distributed, while those in subset 4 make more pronounced contributions to the model’s overall performance.

In this study, we systematically assessed the relative importance of selected features in predicting material properties using SVR models. The experimental results demonstrate that the SVR model exhibits exceptional predictive accuracy, confirming its effectiveness and superiority for material property prediction tasks. To quantitatively analyze the contribution of each feature to the model’s performance, we employed permutation testing to evaluate feature importance. The importance scores for each feature were normalized, ensuring values ranged from 0 to 1, with the sum of all feature importance scores equal to 1. The normalized importance scores reflect the relative contribution of each feature to the model’s predictive performance, with higher scores indicating a more significant impact. The normalized feature importance of the model results is shown in Figure 8a.

A detailed analysis of the feature importance scores revealed that subset 4 had the highest average importance score of 0.0103 among all subsets, indicating its superior performance. Moreover, subset 4 occupied 9 out of the top 20 positions in the feature importance ranking (Figure 8b), outperforming the other subsets, with subset 1 following closely behind, occupying 7 positions. Further analysis indicated that the variance in feature importance rankings for subset 4 and subset 1 was significantly lower than for the other subsets (Figure 8d), suggesting that the contributions of these subsets were more stable. The features in subset 1 primarily relate to the atomic properties of metal elements and their crystal structures, while subset 4 focuses on the microscopic performance characteristics of metal nitrides. This result highlights a stronger correlation between the model’s predictive performance, the fundamental properties of metal crystals, and the microscopic characteristics of metal nitrides, with the latter playing a pivotal role in hardness prediction.

In contrast, the features from subset 2 and subset 3 exhibited significantly lower importance scores, both in terms of average importance, the proportion of top 20 ranked features, and variance in importance scores, especially for subset 3, which showed markedly lower importance compared to the other subsets. This can be attributed to the fact that subset 3 features are primarily related to the macroscopic properties of elemental substances, which are less correlated with the performance of the final ternary nitrides. In comparison, subset 2 includes features related to periodic properties, ionization energy, and electronegativity, which contribute to the performance of ternary nitrides but have a lesser overall impact on predictive performance than those from subset 1 and subset 4.

In conclusion, feature selection should prioritize characteristics that reflect the microscopic properties of metal nitrides, rather than being limited to the atomic structure and crystal attributes of metal elements. This strategy not only enhances the predictive accuracy of the model but also provides crucial scientific insight into the intrinsic relationship between material properties and their microstructure, thereby advancing research in material design and performance optimization.

In this study, we employed an SVR model to investigate the relative importance of selected features in influencing the model’s performance. The experimental results demonstrated that the SVR classifier achieved high detection accuracy, underscoring its effectiveness in predictive tasks. To further quantify the contribution of individual features to the model’s performance, we utilized a permutation importance test to rank the features. The ranking results, illustrated in Figure 8b, indicate that Feature 1 and Feature 8 ranked the highest among all features, highlighting their dominant role in the prediction process and their substantial contribution to enhancing the model’s detection accuracy.

In addition to evaluating the importance of individual features, we conducted a systematic analysis of feature interdependence, as correlations between features can significantly impact the model’s predictive capabilities. To quantify the linear relationships between features, we employed the Pearson correlation coefficient (PCC). This metric evaluates the strength of the linear relationship between two variables, as expressed by Equation (29), where ${\bar{f}}_{1}$ and ${\bar{f}}_{2}$ represent the mean values of features $f_{1}$ and $f_{2}$, respectively. The Pearson correlation coefficient r ranges from −1 to 1, with values closer to −1 or 1 indicating stronger negative or positive linear correlations, respectively. A value of r = 0 signifies no linear correlation between the features.

(29) $$r=\frac{\left\{\sum \left(f_{1}-{\bar{f}}_{1})(f_{2}-\bar{f_{2}}\right)\right\}}{\left\{\sqrt{\sum {\left(f_{1}-{\bar{f}}_{1}\right)}^{2}}\sqrt{\sum {\left(f_{2}-{\bar{f}}_{2}\right)}^{2}}\right\}}$$

**Figure 9:**
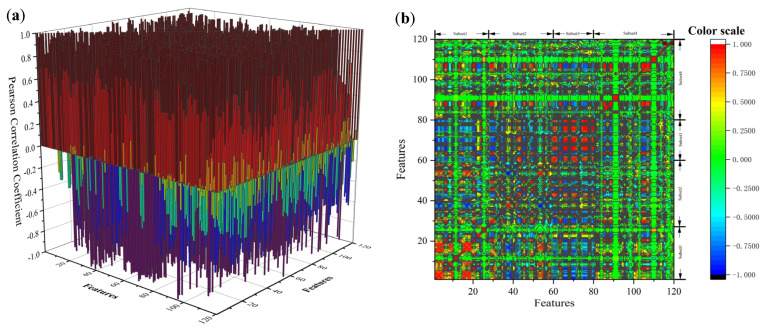
(**a**) The 3D plot of the pearson correlation coefficients between all features. (**b**) The 2D projection plot of the pearson correlation coefficients between all features.

Figure 9a,b present a visualization of the Pearson correlation coefficients between features, offering a clear depiction of their interrelationships. The analysis revealed significant correlations between certain features, suggesting the need to mitigate redundancy during the feature selection process. Redundant features can increase the complexity of the model and compromise its stability and generalizability. These findings emphasize the importance of addressing feature interdependencies to streamline the model structure and improve performance. The analysis of feature correlations thus provides critical theoretical insights for optimizing the model and enhancing its predictive capabilities.

From Figure 9a,b, it is evident that the features within subset 4 exhibit significantly lower correlations with those in other subsets. This can be attributed to the distinctive nature of subset 4 features, which are closely tied to the specific properties of metal nitrides. In contrast, the features of other subsets predominantly represent attributes related to elemental metals or individual elements. Metal nitrides exhibit unique physical and chemical characteristics at the microstructural and mesostructural levels, distinguishing them from the atomic structures and crystalline properties of elemental metals. Consequently, the structural and property-level dissimilarities between subset 4 and the other subsets result in reduced overlap and, thus, lower inter-subset correlations. This observation underscores the indispensable importance of subset 4 features in the model, particularly for tasks involving the prediction of properties specific to metal nitrides. The distinctiveness of subset 4 features is especially crucial for advancing our understanding of the unique characteristics of metal nitrides.

In contrast, features in subset 3 demonstrate higher correlations with those in other subsets, likely due to their focus on the macroscopic properties of materials. These macroscopic attributes often emerge as aggregated expressions of microstructural and mesostructural characteristics. For example, subset 3 features provide a holistic representation of material properties, integrating information from multiple scales. This integrative capacity accounts for the stronger correlations observed between subset 3 and the other subsets. Further analysis reveals that subset 3 plays a pivotal role in describing the overall performance of materials, while its high correlation with other subsets highlights the intrinsic connections between macroscopic properties and the underlying microstructural and mesostructural characteristics. This finding validates the model’s capability to incorporate multi-scale features effectively, thereby establishing a robust foundation for comprehensive material property prediction.

### 5.2. Different Feature Subset Models

In traditional applications of materials machine learning, the inherent constitutive relationships between the physicochemical properties of materials, and their macroscopic mechanical performance are often excluded from modeling processes. As a result, materials design is frequently reduced to a purely mathematical or statistical optimization problem. However, such simplifications overlook the intrinsic mechanisms and complexities inherent to materials science. To address this limitation, the present study identifies and integrates a diverse set of feature variables that link materials design with their microstructural and mesoscale features as well as their thermodynamic and kinetic properties. By employing mathematical modeling, these physicochemical attributes are mapped onto a high-dimensional feature space within artificial intelligence frameworks, thereby playing a pivotal role in performance prediction.

The acquisition of raw input data in materials science is a time-intensive and costly endeavor, making data collection particularly challenging. This constraint results in limited sample spaces, which pose a significant bottleneck for the advancement of materials ML research. To overcome this challenge, this study introduces a high-dimensional feature space strategy, which not only effectively enriches the information content of available data but also improves data quality. This approach offers a novel solution to small dataset problems while substantially enhancing the model’s capability to predict complex material properties.

To systematically investigate the influence of different feature subsets on the generalization ability of the SVR model, the study conducted a comprehensive analysis of all feature subsets. Specifically, by comparing the model’s performance during training and testing with and without the inclusion of different feature subsets, the contribution of specific variables to the model’s performance was assessed. The feature subsets used in the model are summarized in Table 2. The modeling process adhered strictly to the previously outlined methodologies to ensure the repeatability of the experiments and the reliability of the conclusions. This analysis provides critical insights for optimizing feature selection and advancing model performance, offering a robust foundation for future materials design efforts.

In addition to significantly enhancing the model’s generalization capability, this approach offers an innovative solution to address the issue of small sample datasets. Traditionally, increasing the dataset size is a common strategy to mitigate small sample problems. However, in materials science, this process not only depends on the richness of the raw input data but also requires additional information obtained through standardized experiments, such as microstructural features. The time-consuming and costly nature of experimental processes, along with variations in experimental conditions that can compromise data consistency and quality, limits the applicability of this strategy in practice.

To overcome these challenges, this study introduces high-reliability variables related to the material itself, thereby significantly expanding the dimensionality of the dataset without relying on large-scale experimental data. Specifically, these variables provide more diverse and refined information for each sample, effectively broadening the feature space and enriching the hierarchical representation of the data. This strategy also mitigates the impact of experimental errors on data quality, improving data consistency and reliability. Moreover, by modeling within a high-dimensional feature space, the approach reduces the standard deviation during model training, alleviating the overfitting caused by insufficient data and significantly enhancing the robustness and reliability of the model’s predictions.

This method transcends the limitations of traditional data augmentation techniques, offering a novel approach to addressing small sample issues and holding significant theoretical and practical value in improving model performance and optimizing material design.

**Figure 10:**
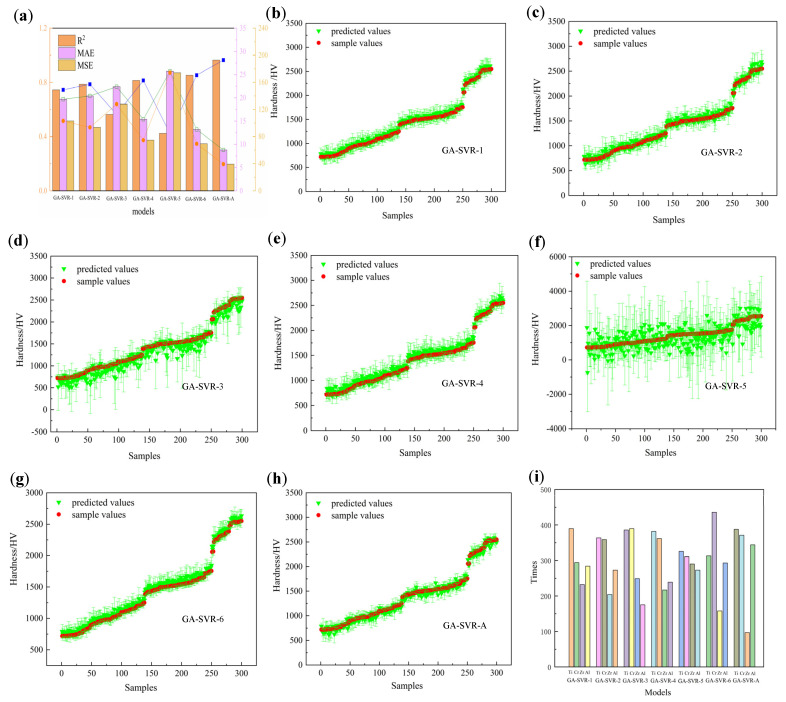
(**a**) The MSE, MAE, and R^2^ of all models. (**b**–**h**) Ten prediction results of all models. (**i**) The times of element distribution of all models.

The ultimate goal of the GA-SVR model developed in this study is to apply it to the design of novel materials. To explore the impact of incorporating different feature parameters on material design, we employed GA-SVR models based on various feature sets for the design of ternary metal nitride stainless steel. In these models, feature subsets were used as the objective function for the GA. By testing multiple GA-SVR models, we compared their performance across 10 test sets, evaluating metrics such as MAE, MSE, and R^2^, with the results presented in Figure 10a. All SVR models achieved R^2^ values greater than 87%, indicating that the models performed well across all feature combinations.

Further analysis of performance metrics, including MAE, MSE, and R^2^ (Figure 10b), revealed that the GA-SVR-A model demonstrated significantly better fitting than the other models, with the most concentrated element distribution. This suggests that the contribution of feature subsets to the model is not simply a linear sum but rather a result of their mutual coupling and synergistic interaction. Additionally, models incorporating three feature subsets (such as GA-SVR-A) outperformed those with only two feature subsets, further confirming the importance of feature coupling in enhancing model performance. This also underscores the critical role of interactions between feature subsets in improving predictive accuracy.

Moreover, the comparison of model performances indicates that GA-SVR-6 and GA-SVR-4 follow GA-SVR-A in terms of fitting accuracy. The shared characteristic of these models is that they include all three feature subsets and specifically feature subset 1. This finding aligns closely with the analysis of feature subset importance and correlation in Section 5.1, providing further evidence that the coupling between feature subsets is key to improving model prediction accuracy. Consequently, this study suggests that in material design tasks, feature selection should focus not only on the individual effects of features but also on the interactions between feature subsets.

In traditional experimental designs, the formation process of substitutional solid solutions is also considered, involving the substitution of different metal atoms in the lattice. This process is influenced by factors such as diffusion, temperature, lattice defects, and atomic size differences. In Ti-, Cr-, Zr-, and Al-based multi-metal nitrides, the kinetics of substitutional solid solution formation determine the microstructure of the nitrides. For example, the substitution of Ti and Zr atoms in the lattice can enhance the lattice stability, thus affecting the material’s hardness and toughness. The diffusion rate is a key parameter controlling the formation of substitutional solid solutions. Additionally, lattice defects such as vacancies and dislocations serve as fast channels for diffusion, accelerating the migration of solute atoms and promoting the formation of substitutional solid solutions. However, these defects may also introduce stress concentration, leading to increased brittleness in the material. These issues are typically considered by designers based on experience.

As shown in Figure 10a, the GA-SVR-3 model exhibits slightly lower MAE, MSE, and R^2^ values compared to GA-SVR-4, but these values are still higher than those of GA-SVR-4. Notably, the MAE, MSE, and R^2^ values of GA-SVR-6 are significantly better than those of the other five models, except for GA-SVR-A. This improvement can be attributed to the inclusion of feature variables in subset 2, which consist of atomic structure properties (including atomic structure, atomic energy, and chemical bonds) as well as thermodynamic and kinetic properties such as Gibbs free energy and entropy. These properties govern the structural characteristics, phonon density of states, and Debye temperature of multi-metal nitrides, thereby influencing their macroscopic mechanical properties. Furthermore, these thermodynamic properties affect the stability of new structures and the rate of diffusion. However, due to the steady-state deposition of coatings in multi-arc ion plating experiments, the influence of kinetics is greatly reduced. Nevertheless, the unique atomic properties collectively shape the microstructure and chemical stability of nitrides, thereby determining the material’s mechanical properties. For example, Ti, Cr, Zr, and Al, all transition metals, have distinct electronic configurations and atomic radii. Ti and Zr share similar electronic structures (d^2^sp^3^ hybridization), enabling them to form strong metal–nitrogen bonds in nitrides. Cr, with d^3^sp^3^ hybridization, possesses higher electronic density, which facilitates the formation of stable nitride structures. Al, though not a transition metal, forms stable chemical bonds with nitrogen due to its sp^2^ hybridization. In terms of atomic energy, the differences in atomic energy between Ti, Cr, Zr, and Al result in different behaviors in nitrides. Ti and Zr have higher melting and boiling points, indicating better stability at high temperatures. Although chromium has a lower melting point and boiling point, its higher hardness and corrosion resistance make it an ideal component for enhancing the hardness of nitrides. Aluminum’s low melting point and high thermal conductivity improve the processing performance of nitrides. Regarding chemical bonds, the strength and nature of metal–nitrogen bonds play a crucial role in the hardness of nitrides. The strong metal–nitrogen bonds in Ti and Zr contribute to higher hardness and wear resistance, while Cr forms moderate-strength bonds that balance hardness and toughness. Aluminum forms relatively weaker bonds, but its inclusion improves the oxidation resistance and thermal stability of the nitrides.

In contrast, the GA-SVR-5 model shows the lowest performance. The feature subset 3 focuses on the macroscopic properties of each elemental material. In traditional experiments, macroscopic attributes such as hardness are often linearly correlated to estimate the total contribution of the final result. However, in actual material preparation, many factors contribute to the final result, and the relationship with macroscopic properties is often nonlinear. Compared to this, SVR models can significantly reduce preparation complexity. However, macroscopic features usually provide an overall description of material properties and may not capture the microscopic mechanisms that influence material hardness. This is likely the reason for the poorer performance of the GA-SVR-5 model.

From the perspective of element distribution (as shown in Figure 10i), it is observed that models incorporating subset 2 feature subsets show a more concentrated element distribution, with Ti and Cr elements appearing much more frequently than others. This indicates a complex relationship between the hardness of metal nitrides and their lattice distortions, particularly with respect to chemical bonds and crystal structures. Further analysis reveals that elements’ electronegativity, ionization energy, and thermodynamic properties make significant positive contributions to the hardness prediction of metal nitrides. These physicochemical properties directly affect the strength of metal nitrides, mainly through their role in modulating atomic bonding forces and influencing crystal structure stability. Specifically, elements such as Ti and Cr, with high electronegativity and good thermodynamic stability, contribute to nitrides with higher hardness and stronger resistance to deformation. Thus, the electronegativity, ionization energy, and related thermodynamic properties of these elements play a crucial role in predicting the hardness of metal nitrides, revealing the intrinsic connection between the microstructure and macroscopic mechanical properties of these materials.

### 5.3. Analysis of Prediction Results and Experimental Validation

In this study, the GA-SVR-A model was used to predict hardness, and the predictions were compared with experimental data. The test results demonstrated that the GA-SVR-A model significantly outperformed other models in terms of MAE, MSE, and R^2^. The model’s superiority lies not only in the linear combination of the four feature subsets but also in its ability to provide complementary information between subsets. This reduces the model’s over-reliance on any particular feature set, thereby enhancing its generalization capability.

For hardness prediction, the GA-SVR-A model was tested with 100 predictions, resulting in an average predicted hardness of 2960.02 HV. Notably, 82 of the predictions exceeded the maximum hardness value in the dataset. To further explore the relationship between element molar ratios and hardness in the predictive model, we selected the element ratio from the first prediction as a baseline and performed variance analysis on the remaining predictions against this baseline. This approach quantifies the differences in element composition across predictions, allowing for an assessment of the impact of element molar ratios on hardness prediction. The specific formula used is shown in Equation (30).

(30) $$VM=\frac{1}{n}{\sum \left(x_{i}-x_{1}\right)}^{2}\;\;\;\;\;\;\left(i=2,\ldots \ldots ,100\right)$$

Here, VM represents the variance between element molar ratios, *x_i_* denotes the molar ratio of elements in the *i*-th prediction, and *x*_1_ represents the molar ratio in the first prediction.

To mitigate the impact of preparation accuracy and measurement errors on the model’s predictions, clustering analysis was performed on samples with hardness predictions exceeding 3200 HV. The specific steps are as follows:

(1) Calculation of distances between samples: First, the distances between the 19 samples with hardness values greater than 3200 HV were calculated. The average of these distances was then obtained using Equation (31) to provide an overall distribution of the sample points.
  (31) $$R_{nei}=\frac{1}{4n}\sqrt{{\left(x_{i}-x_{j}\right)}^{2}+(y_{i}-y_{j}{)}^{2}}\begin{array}{c} \left(i,j=1,2,3,\ldots 19\;and\;i\neq j\right) \end{array}$$
(2) Clustering analysis: The DBSCAN algorithm was employed to cluster the 19 sample points. The neighborhood radius was set to *R_nei_*, with the minimum sample number *MinPts* set to 3. If a point had a neighborhood sample count greater than *R_nei_* but fewer than MinPts, it was considered a noise point and excluded from the clustering process. This method effectively identifies and removes outliers, thereby enhancing the accuracy of the clustering analysis.
(3) Objective function construction and gradient descent optimization: An objective function was established to compute the centroid coordinates (a,b) and the minimum radius r_area_ for each cluster, with the clustering areas labeled as Area 1, Area 2, and Area 3. This approach effectively extracts core data features within the clustering regions.

(32) $$J(a,b,r_{area})=\sum_{i=1}^{19}{\left({\left(x_{i}-a\right)}^{2}+{\left(y_{i}-b\right)}^{2}-{r_{area}}^{2}\right)}^{2}$$

**Figure 11:**
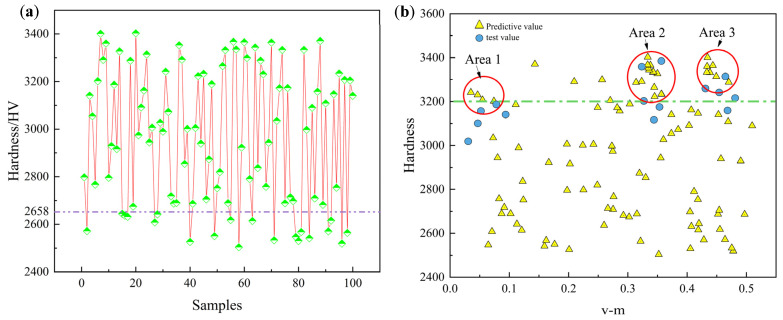
(**a**) Ten prediction results of the GA-SVR-A model. (**b**) Predicted and experimental values of the GA-SVR-A model.

Within the clustering regions, it was assumed that the relationship between material hardness and molar ratio followed the functional relationship shown in Figure 11b. Therefore, the element ratios extracted within each neighborhood should correspond to the hardness values within that region. Based on this assumption, the value ranges for the three clustering areas are shown in Table 1. To validate the model’s predictive accuracy, five experimental sets were conducted within each of the three areas, and the corresponding molar ratios were recorded (see Table 3). The experimental results indicated that Area 2 contained the most samples, followed by Area 1, with Area 3 containing the fewest samples.

To validate the model’s predictions, five sets of experiments were conducted within each of the three regions, with molar ratios provided in Table 4. In all validation experiments, all sample points in Area 2 fell within the region. In contrast, only three sample points from Area 3 and one from Area 1 were found within their respective regions. Subsequently, all fifteen samples were input into the trained GA-SVR-A model, and the results showed that the samples from Area 2 exhibited the smallest prediction errors. This suggests that the more prediction points within a region, the closer the relationship between the element molar ratio and hardness aligns with the functional relationship shown in Figure 11b. Conversely, the fewer the prediction points within a region, the greater the deviation from the relationship.

**Figure 12:**
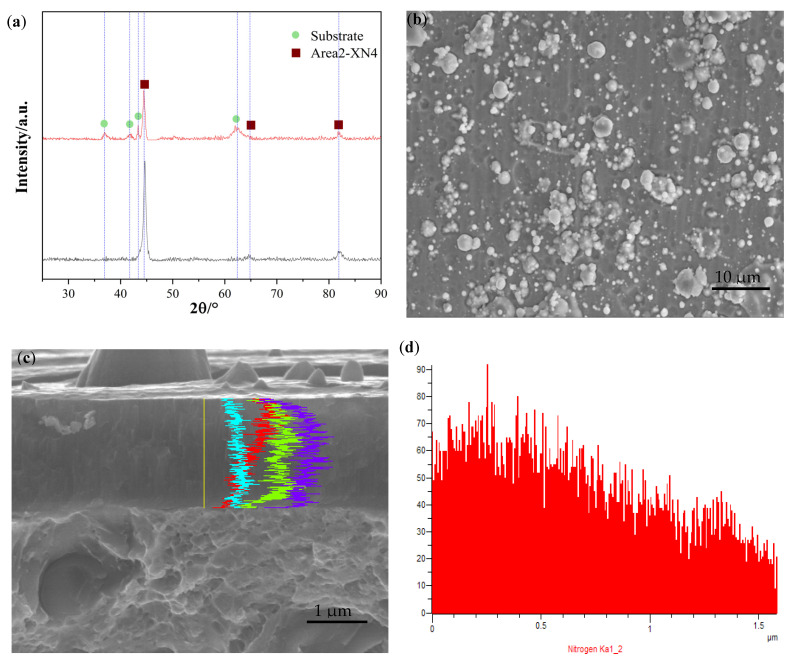

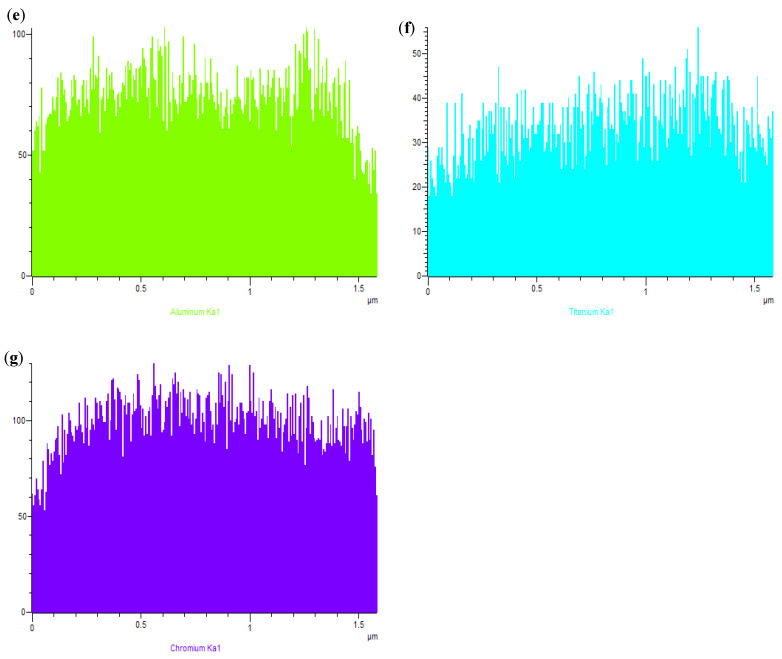
(**a**) The XRD of Area2-XN4. (**b**) TEM image of Area2-XN4. (**c**–**g**) EBSD of Area2-XN4.

The XRD spectrum of the sample Area2-XN5 also shows four main diffraction peaks, as illustrated in Figure 12a, corresponding to the (111), (200), and (220) planes, and the CrTiAl alloy phase diffraction peak, all exhibiting typical face-centered cubic lattice characteristics. From the surface morphology in Figure 12b, it is evident that the coating surface is continuous; dense; and free from peeling, porosity, or cracking. The alloy phase is observed to originate from droplets within the coating. The distribution of elements in the coating is shown in Figure 12c,d,f,g. These results are in line with our original design concept.

## 6. Conclusions

In this work, a novel approach is presented for the design of multicomponent metal nitride hard coatings. The method begins with the electronic structure of high-hardness unary metal nitrides, establishing a compositional search space based on the experimental conditions. This search space is further constrained by the crystal structure and strengthening mechanisms, and a target function is defined for the model. An improved GA combined with SVR is then employed to design a high-hardness multicomponent metal nitride, which is experimentally validated through hardness testing.

The introduction of feature sets enhances the model’s ability to consider strengthening mechanisms across micro-, macro-, and mesoscopic dimensions. To investigate the influence of different dimensional strengthening mechanisms on hardness enhancement, the study reveals that subset 1 has a significantly higher impact than other subsets. This is attributed to the inclusion of higher-dimensional features, which improve the model’s ability to capture complex relationships between variables. Additionally, the inclusion of such high-dimensional features is particularly beneficial for systems with small datasets, where traditional methods may be less effective.

Experimental validation confirms that the predicted optimal parameters align closely with the final performance of the coatings, demonstrating the robustness and accuracy of the proposed model. The use of clustering analysis minimizes experimental and data errors, further enhancing the reliability of the predictions. Of the 100 predictions made by the model, 82 were higher than the maximum hardness in the dataset, and the prediction accuracy for the best sample was 91.6%.

Despite these achievements, the approach has limitations that warrant future investigation. The dataset of 233 entries is relatively small, potentially limiting the model’s ability to capture the full compositional variability of multicomponent nitrides. Additionally, the model’s reliance on multi-arc ion plating for validation may restrict its generalizability to other synthesis methods, such as magnetron sputtering. Furthermore, feature engineering based on solid solution strengthening theory assumes specific physical mechanisms, which may not fully account for all interactions in complex nitride systems. Future work should focus on expanding the dataset with diverse nitride compositions, testing the model across various deposition techniques, and refining feature engineering by incorporating additional physical models to enhance predictive robustness.

## Figures and Tables

**Table 1:** AUCS for detection performance with all features by ten times.

Times	1	2	3	4	5	6	7	8	9	10
AUC	0.945	0.901	0.899	0.915	0.963	0.959	0.891	0.932	0.964	0.941

**Table 2:** The feature subsets used in the model.

Model	Feature Subset
GA-SVR-1	Subset 1 + subset 4
GA-SVR-2	Subset 2 + subset 3 + subset 4
GA-SVR-3	Subset 2 + subset 4
GA-SVR-4	Subset 1 + subset 3 + subset 4
GA-SVR-5	Subset 3 + subset 4
GA-SVR-6	Subset 1 + subset 2 + subset 4
GA-SVR-A	All sets (subset 1 + subset 2 + subset 3 + subset 4)

**Table 3:** Range of metal content proportions observed in the three areas of Figure 11.

Area	Ti	Al	Cr	Zr
Area 1	0	0.0855~0.125	0.106~0.153	0.218~0.290
Area 2	0.0865~0.115	0.189~0.269	0.187~0.215	0
Area 3	0.218~0.255	0.185~0.166	0.147~0.181	0

**Table 4:** Predicted and experimental values of the GA-SVR-A model.

Area	Experimental Sample	Element Ratio	Test Strength (HV)	Model Prediction Strength (HV)	Prediction Accuracy
Area 1	Area1-XN1	Al_0.08_Cr_0.13_Zr_0.22_N_0.5_	3189	3230	98.73%
	Area1-XN2	Al_0.06_Cr_0.06_Zr_0.13_N_0.5_	3160	3164	99.87%
	Area1-XN3	Al_0.085_Cr_0.15_Zr_0.265_N_0.5_	3140	3244	96.79%
	Area1-XN4	Al_0.10_Cr_0.12_Zr_0.22_N_0.57_	3100	3253	95.30%
	Area1-XN5	Al_0.10_Cr_0.145_Zr_0.26_N_0.5_	3017	3241	93.09%
Area 2	Area2-XN1	Ti_0.1q_Al_0.19_Cr_0.21_N_0.5_	3356	3293	98.12%
	Area2-XN2	Ti_0.09_Al_0.20_Cr_0.21_N_0.5_	3207	3348	95.79%
	Area2-XN3	Ti_0.09_Al_0.17_Cr_0.18_N_0.57_	3178	3362	94.53%
	Area2-XN4	Ti_0.095_Al_0.20_Cr_0.2_N_0.5_	3385	3271	96.63%
	Area2-XN5	Ti_0.11_Al_0.19_Cr_0.2_N_0.5_	3115	3398	91.67%
Area 3	Area3-XN1	Ti_0.21_Al_0.26_Cr_0.28_N_0.25_	3314	3241	97.79%
	Area3-XN2	Ti_0.13_Al_0.04_Cr_0.08_N_0.5_	3260	3292	99.03%
	Area3-XN3	Ti_0.15_Al_0.27_Cr_0.33_N_0.25_	3243	3287	98.66%
	Area3-XN4	Ti_0.23_Al_0.10_Cr_0.17_N_0.5_	3220	3291	97.84%
	Area3-XN5	Ti_0.23_Al_0.08_Cr_0.17_N_0.5_	3162	3282	96.34%

## Data Availability

The original contributions presented in this study are included in the article/Supplementary Materials. Further inquiries can be directed to the corresponding author.

## Supplementary Materials

The following supporting information can be downloaded at: https://www.mdpi.com/article/10.3390/ma18153478/s1, Table S1. The elements of the set subset-N. Table S2. All the features and the details of their subsets. Table S3. List of Abbreviations.
