# Supplementary material for: Machine Learning-Driven Design and Optimization of Multi-Metal Nitride Hard Coatings via Multi-Arc Ion Plating Using Genetic Algorithm and Support Vector Regression
Source: Materials (Basel). 2025 Jul 24;18(15):3478. doi: 10.3390/ma18153478 (PMC12347041; doi:10.3390/ma18153478)
Supplement: Supplementary file 1 [file materials-18-03478-s001.zip › materials-3641085-supplementary.pdf]

materials

MDPI

Machine Learning Drived Design and Optimization of Multi-Metal Nitride Hard Coatings via Multi-Arc Ion Plating Using Genetic Algorithm and Support Vector Regression

Yu Gu [1,2], Jiayue Wang [4],ZhangYu [1,2], Bushi Dai [1,2], Li Yu [3],Liu Jing [7], Zhang Jun * [1], Guangchao Liu [1], Bao Li [5] *and* Rihuan Lu [6]

[1] College of Mechanical Engineering, Shenyang University, Shenyang 110044, China
[2] Laboratory of Research and Application of Multiple Hard Films, Shenyang University, Shenyang 110044, China
[3] College of Intelligence and Informatics, Shenyang University, Shenyang 110044, China
[4] Informatization Office, Shenyang University, Shenyang 110044, China
[5]School of Mechanical and Electrical Engineering, Qiqihar University, Qiqihar 161000, China
[6]National Engineering Research Center for Equipment and Technology of Cold Rolled Strip, Yanshan University, Qinhuangdao, 066004, Hebei, China
[7]Department of Radiology, The First Hospital of China Medical University,110001 Shenyang, China
* Correspondence: author. Tel: +86 18804013087; neu_syu_tougao@hotmail.com (Jun Zhang)

Table S1. The elements of the set subset-N.

Description	TiN	AlN	CrN	CrN_2	Cr_3N_2	Cr_3N_4	ZrN	Zr_3N_4	Zr_3N
Energy Above Hull（eV/atom）	0.97	0.172	0	0.924	0.114	0.163	0.322	0.043	1.559
Band Gap（eV）	0	4.42	0	0	0	0	0	0.56	0
Predicted Formation Energy（eV/atom）	0.97	-1.508	-0.749	0.424	-0.486	0	-1.654	-1.81	0.501
Number of Atoms	8	8	8	12	80	28	8	56	4
Density（$g·cm^{-3}$）	11.98	4.04	5.96	5.06	6.39	4.47	5.62	5.73	6.81
lattice constant a (pm)	4.95	4.07	4.19	4.72	9.15	6.8	4.99	9.14	4.12
lattice constant b (pm)	4.95	4.07	4.19	4.72	9.15	6.8	4.99	9.14	4.12
lattice constant c (pm)	4.95	4.07	4.19	4.72	9.15	6.8	4.99	9.14	4.12
α（°）	90	90	90	90	90	90	90	90	90
β（°）	90	90	90	90	90	90	90	90	90
γ（°）	90	90	90	90	90	90	90	90	90
Volume	121.08	67.37	73.61	104.94	765.27	314.83	124.31	764.26	70.11
International Number	225	225	225	225	199	220	216	227	221
average x	0.11290323	0.1129032	0	0.056452	0.701279781	0.355041303	0.066037736	0.764447263	0
average y	0	0	0.106061	0.056452	0.371622151	0.046473848	0.066037736	0.299800798	0.433962264
average z	0	0	0	0.169355	0.540096905	0.362617061	0.066037736	0.419366182	0.433962264
enthalpy vaporization (kJ mol^{-1})	27.09	26.71	26.26	27.21333	25.688	26.975	22.2	25.64	23.03
Enthalpy of fusion (kJ mol^{-1})	5.76282566	5.7628257	5.762826	5.291979	5.595419	5.500236762	5.762825659	5.677697982	4.675254392

Table S2. All the features and the details of their subsets.

Subset sertial number	statistical method	Serial number	Description
Subset1	$\bar{x}=\sum c_i x_i$	1	radii atomic (empirical) (pm)
		2	radii covalent (empirical) (pm)
		3	Molecular single bond covalent radius
		4	Molecular double bond covalent radius
		5	van der Waals radius
		6	radii metal (Waber) (A)
		7	volume atom (Villars, Daams) (pm^3)
		8	lattice constant a (pm)
		9	lattice constant b (pm)
		10	lattice constant c (pm)
		11	α (°)
		12	β (°)
		13	γ (°)
		14	atomic Volume
	$\delta_x=\sqrt{\sum c_i(1-x_i/\bar{x})}$	15	radii atomic (empirical) (pm)
		16	radii covalent (empirical) (pm)
		17	Molecular single bond covalent radius
		18	Molecular double bond covalent radius
		19	van der Waals radius
		20	radii metal (Waber) (A)
		21	volume atom (Villars, Daams) (pm^3)
		22	lattice constant a (pm)
		23	lattice constant b (pm)
		24	lattice constant c (pm)
		25	α (°)
		26	β (°)
		27	γ (°)
		28	atomic Volume

Continued Table S2: All the features and the details of their subsets.

Subset sertial number	statistical method	Serial number	Description
Subset2	$\bar{x}=\sum c_i x_i$	29	atomic number
		30	atomic weight (10^{-3} kg)
		31	main group or subgroup element
		32	Effective nuclear charges
		33	valence electron number
		34	Number of rows in the periodic table
		35	Number of columns in the periodic table
		36	outermost electron number
		37	electronegativity (Pauling)
		38	electronegativity (Allred Rochow)
		39	energy ionization first (kJ mol^{-1})
		40	energy ionization second (kJ mol^{-1})
		41	energy ionization third (kJ mol^{-1})
		42	work function (eV)
		43	enthalpy vaporization (kJ mol-1)
		44	Enthalpy of fusion (kJ mol-1)
	$\delta_x=\sqrt{\sum c_i(1-x_i/\bar{x})}$	45	atomic number
		46	atomic weight (10^{-3} kg)
		47	main group or subgroup element
		48	Effective nuclear charges
		49	valence electron number
		50	Number of rows in the periodic table
		51	Number of columns in the periodic table
		52	outermost electron number
		53	electronegativity (Pauling)
		54	electronegativity (Allred Rochow)
		55	energy ionization first (kJ mol^{-1})
		56	energy ionization second (kJ mol^{-1})
		57	energy ionization third (kJ mol^{-1})
		58	work function (eV)
		59	enthalpy vaporization (kJ mol-1)
		60	Enthalpy of fusion (kJ mol-1)

Continued Table S2: All the features and the details of their subsets.

Subset sertial number	statistical method	Serial number	Description
Subset3	$\bar{x} = \sum c_i x_i$	61	Melting Point （K）
		62	Boiling Temperature （K）
		63	Density（g·cm^{-3}）
		64	Molar volume
		65	modulus bulk (GPa)
		66	modulus rigidity (GPa)
		67	modulus Young (GPa)
		68	Poisson's ratio
		69	Mineral hardness
		70	Brinell hardness(MN m^{-2})
	$\delta_x = \sqrt{\sum c_i (1 - x_i / \bar{x})}$	71	Melting Point （K）
		72	Boiling Temperature （K）
		73	Density（g·cm^{-3}）
		74	Molar volume
		75	modulus bulk (GPa)
		76	modulus rigidity (GPa)
		77	modulus Young (GPa)
		78	Poisson's ratio
		79	Mineral hardness
		80	Brinell hardness(MN m^{-2})

Continued Table S2: All the features and the details of their subsets.

Subset sertial number	statistical method	Serial number	Description
Subset4	$\bar{x}=\sum c_i x_i$	81	XN-Energy Above Hull（eV/atom）
		82	XN-Band Gap（eV）
		83	XN-Predicted Formation Energy（eV/atom）
		84	XN-Number of Atoms
		85	XN-Density（g·cm^{-3}）
		86	XN-Space group number
		87	XN-lattice constant a (pm)
		88	XN-lattice constant b (pm)
		89	XN-lattice constant c (pm)
		90	XN- α（°）
		91	XN- β（°）
		92	XN- γ（°）
		93	XN-Volume
		94	XN-International Number
		95	XN-average x
		96	XN-average y
		97	XN-average z
		98	XN-enthalpy vaporization (kJ mol-1)
		99	XN-Enthalpy of fusion (kJ mol-1)
	$\delta_x=\sqrt{\sum c_i(1-x_i/\bar{x})}$	100	XN-Energy Above Hull（eV/atom）
		101	XN-Band Gap（eV）
		102	XN-Predicted Formation Energy（eV/atom）
		103	XN-Number of Atoms
		104	XN-Density（g·cm^{-3}）
		105	XN-Space group number
		106	XN-lattice constant a (pm)
		107	XN-lattice constant b (pm)
		108	XN-lattice constant c (pm)
		109	XN- α（°）
		110	XN- β（°）
		111	XN- γ（°）
		112	XN-Volume
		113	XN-International Number
		114	XN-average x
		115	XN-average y
		116	XN-average z
		117	XN-enthalpy vaporization (kJ mol-1)
		118	XN-Enthalpy of fusion (kJ mol-1)
		119	XN-entropy mix
		120	XN-Enthalpy mix

Table S3. List of Abbreviations:.

Abbreviations	The full name
N atoms	Nitrogen atoms
FCC	Face-centered cubic crystal structure
ML	Machine learning
GA-SVR	Genetic algorithm-support vector regression
GA	Genetic algorithm
SVR	Support vector regression
Ti	Titanium
Al	Aluminum
Cr	Chromium
Zr	Zirconium
HV	Vickers hardness
